# Editorial: Brain functional analysis and brain-like intelligence

**DOI:** 10.3389/fnins.2024.1383481

**Published:** 2024-03-06

**Authors:** Zhiqiang Tian, Zhengwang Wu, Shihui Ying

**Affiliations:** ^1^School of Software Engineering, Xi'an Jiaotong University, Xi'an, China; ^2^Department of Radiology, UNC-Chapel Hill, Chapel Hill, NC, United States; ^3^Department of Mathematics, School of Science, Shanghai University, Shanghai, China

**Keywords:** brain functional analysis, brain image understanding, EEG signal-based epileptic seizure prediction, brain-machine fusion, complex network, neuroscience-inspired machine learning

## 1 Introduction

The Research Topic “*Brain functional analysis and brain-like intelligence*” belongs to the journals, Frontiers in Neuroscience. The aim of this Research Topic is to establish a bridge between brain functional analysis and brain-like machine intelligence, which will promote the basic theory of AI, as well as the mechanism of brain function.

With the improvement in data collection and computing power, artificial intelligence (AI), represented by deep learning, has been developing rapidly. However, there exist huge gaps between natural data and brain data. It therefore suffers from performance degradation if directly applying a traditional deep learning method to the brain data including image, voxel, and electroencephalography (EEG)-based signal. This brings considerable challenges to the brain-related application. Toward these Research Topics, we strive to offer a thorough understanding of the most recent advancements drawing from all the manuscripts that have been published. The key aspects of this topic can be summarized under the following categories: brain image understanding: registration, recognition, and segmentation; EEG signal-based epileptic seizure prediction; AI for brain science.

## 2 Published papers

### 2.1 Brain image understanding: registration, recognition, and segmentation

With the continuous development and improvement of computer vision technology, AI-based brain image understanding technology including brain image registration, recognition, and segmentation, has increasingly important value in improving the accuracy and efficiency of clinical diagnosis. As an important upstream task of brain image understanding, brain image registration plays an important role on significantly affecting the subsequent downstream process. However, it typically suffers from high model complexity due to the ill-conditioned inverse problem of brain image registration (Fu et al., 2020). Toward this Research Topic, Fang et al. in the article “*Decoupled learning for brain image registration*” decomposed this problem into two simpler sub-problems and adopted two light neural networks to approximate their solutions to reduce the complexity.

With the well registered brain images, its recognition is qualified to be conducted. However, existing brain image recognition methods typically suffer from weak learning ability on shape features. Toward this Research Topic, the article “*STNet: shape and texture joint learning through two-stream network for knowledge guided image recognition*” by Wang et al. proposed a shape and texture joint learning mechanism. In this work, the pyramid-grouped convolution and the deformable convolution are adopted to enhance the shape features.

For medical visual segmentation, the article “*Dual consistent pseudo label generation for multi-source domain adaptation without source data for brain image segmentation*” by Cai et al. proposed a pseudo label generation mechanism for multi-source domain adaptation for brain image segmentation. In this method, a dual consistency constraint including the inter-domain and the intra-domain is presented to guide the generation of the pseudo labels. Beside 2-d segmentation, the 3-d voxels identification is also deserved attention. The article “*Groupwise structural sparsity for discriminative voxels identification*” by Ji, Zhang et al. tackled the absence information of sufficient sample sizes for psychological experiments by proposing a stable hierarchical voting (SHV) mechanism. SHV is enabled to evaluate the quality of spatial random sampling and minimizes the risk of false and missed detection.

### 2.2 EEG signal-based epileptic seizure prediction

In clinical settings, automatic epileptic seizure prediction is crucial to reducing the heavy burden for patients with intractable epilepsy (Zhao et al., 2021). Electroencephalography (EEG) signals record brain activity and provide valuable information about brain dysfunction. But visually evaluating these signals, which is a non-invasive and affordable way to detect seizures, can be time-consuming and subjective. Thus, there's room for improvement. The article “*Epileptic seizure detection with deep EEG features by convolutional neural network and shallow classifiers*” by Zeng et al. exploits the usage of deep learning to achieve automatically detecting seizures as an urgent problem in clinical application. To effectively detect seizures, EEG signals are adopted as input and the combination of deep feature extractor and shallow classifier is proved to be the most effective. Different from this work, in the article “*An effective fusion model for seizure prediction: GAMRNN*”, Ji, Xu, et al. explored the effectiveness of the convolutional attention module toward electroencephalography-based epileptic seizure recognition. In this work, the effectiveness of Lion optimizer is also demonstrated in terms of convergence and the ability to facilitate the recognition performance.

### 2.3 AI for brain science

Brain-machine interfaces (BMI) have developed rapidly in recent years, but still face critical issues such as accuracy and stability (Liu et al., 2020). By mimicking the architecture and functionality of biological nervous systems, neuromorphic computing models emerge as a potential avenue for creating advanced neuroprosthesis with exceptional performance. Qi et al. demonstrates that neuromorphic computing could be a promising method to realize BMI in the article “*Neuromorphic computing facilitates deep brain-machine fusion for high-performance neuroprosthesis*.” The article demonstrates utilizing neuro-morphological computational models to simulate the characteristics of biological neural systems contributes to realizing brain-machine integration and bring new breakthroughs for high-performance and long-term-usable BMI systems.

In recent years, the dynamic behavior of complex networks, especially neural networks, has attracted extensive attention because it can help us understand how the brain processes information, stores memories, and makes decisions (Shine, 2021). The article “*Learning based sliding mode synchronization for fractional order Hindmarsh-Rose neuronal models with deterministic learning*” by Chen et al. proposed a learning based sliding mode control algorithm is proposed by using the deterministic learning (DL) mechanism. With DL mechanism, the synchronization process can be started quickly by recalling the empirical dynamics of neurons. Therefore, fast synchronization effect is achieved by reducing the online computing time.

Inspired by neuroscience, some interpretable machine learning algorithms have been proposed (Lindsay, 2020), such as reinforcement learning mechanisms that simulate brain function. Zhao et al. proposed a neuroscience-inspired reinforcement learning mechanism in the article “*A semi-independent policies training method with shared representation for heterogeneous multi-agent reinforcement learning*.” It is claimed to be the first work to adopt a hard-parameter-sharing scheme to multi-agent reinforcement learning for balancing the conflicting requirements of agents' specialization and fast network convergence.

Moreover, collision prediction algorithms based on the neural model of lobule giant motion detectors (LGMD) is also deserved attention (Zhang et al., 2022). Zheng et al. proposed a LGMD-based model with a binocular structure in the article “*Enhancing LGMD based model for collision prediction via binocular structure*” to address the issue that existing LGMD-based methods are not qualified to learn the valuable depth distance feature. In this work, The depth distance of the moving object is extracted by calculating the binocular disparity facilitating a clear differentiation of the motion patterns.

## 3 Conclusion

Toward the huge gaps between the traditional artificial intelligence and brain science, this Research Topic has gathered considerable original research articles, which made an attempt to establish a bridge between brain functional analysis and brain-like machine intelligence. Among these articles, many brain data-oriented deep learning methods are proposed, toward various downstream tasks such as registration, recognition, segmentation, and detection, which have a vital significance to the clinical application. Moreover, AI for brain science is also discussed, such as brain-machine interfaces and complex neural network construction of brain. These works all contribute positively to reducing the gaps.

## Author contributions

ZT: Writing – original draft, Writing – review & editing. ZW: Writing – review & editing. SY: Writing – review & editing.
